# Predictive factors of stigma in stroke patients based on logistic regression and decision tree mode

**DOI:** 10.12669/pjms.41.5.9946

**Published:** 2025-05

**Authors:** Wenwen Ma, Kunjuan Jing, Ruotong Zhang, Xuefei Li, Zheng Li

**Affiliations:** 1 Wenwen Ma, Ear, Nose and Throat Department, School of Nursing, Hebei University, Baoding, Hebei, China. Baoding No.1 Central Hospital, Baoding, Hebei, China; 2 Kunjuan Jing, School of Nursing, Hebei University, Baoding, Hebei, China; 3 Ruotong Zhang, Department of Thoracic Surgery, School of Nursing, Hebei University, Baoding, Hebei, China. Baoding No.1 Central Hospital, Baoding, Hebei, China; 4 Xuefei Li, Operating Room, Affiliated Hospital of Hebei University, Baoding, Hebei, China; 5 Zheng Li, Department of Nursing, Baoding No.1 Central Hospital, Baoding, Hebei, China

**Keywords:** Decision tree model, Logistic regression model, Sense of stigma, Stroke

## Abstract

**Objective:**

Logistic regression and decision tree model were used to analyze the predictive factors of stigma in stroke patients, and to explore the application value of the two models.

**Methods:**

This was a retrospective study. The data of 342 stroke patients were collected from Baoding No.1 Central Hospital from December 2023 to March 2024. Data were retrospectively retrieved from the hospital information and management system. The regression model and decision tree model of influencing factors of stroke patients’ sense of stigma were established, to analyze the influencing factors of the sense of stigma, and to compare the predictive effects, advantages and disadvantages of the two models.

**Results:**

Logistic regression analysis showed that threat assessment (OR=2.7761) was a risk factor for stigma, while irrelevant cognitive appraisal (OR=0.321), social support (OR=0.098) and resilience (OR=0.438) were protective factors. The results of the decision tree model showed that the patients’ psychological resilience was the most important factor affecting the sense of stigma, followed by social support and threat assessment. The AUC of the decision tree model and Logistic regression model were 0.854 and 0.880, respectively, and the accuracy were 78.7% and 79.6%, respectively.

**Conclusion:**

Threat, irrelevant cognitive appraisal, social support and resilience might be the predictive factors of stigma in stroke patients. The AUC and accuracy of the decision tree model were slightly lower than that of the Logistic regression model.

## INTRODUCTION

Stroke with a disability rate as high as 75%.^1^ While the disease puts them in a certain degree of stigma. A study reveal that stigma is prevalent in stroke patients.^2^ Cognitive appraisal refers to the cognitive process by which an individual realizes the impact of a stressful event on his or her health.^3^ Social support is a key factor in determining the relationship between psychological stress and health.^4^ Social support works with internal factors such as coping styles to influence the individual’s mental health.^5^ A study revealed that stroke patients with greater access to social support had lower levels of stigma^6^, some studies have shown a strong correlation between coping styles and stigma.^7^ Psychological resilience refers to the process of positive adjustment by an individual to achieve physical and mental balance in the face of unfavorable or potentially traumatic events.^8^ Psychological resilience has been found to be associated negatively with stigma. This study used the stress and coped model as a framework, we explored the influencing factors of stigma in stroke patients from a psycho-cognitive perspective based on Logistic regression and decision tree model.

## METHODS

This was a retrospective study. Data from 342 hospitalized stroke patients collected in Baoding No.1 Central Hospital from December 2023 to March 2024 were used. Data were retrospectively retrieved from the hospital information and management system. Collected their various information of all patients, including age, gender, marital status, education level, work status, family residence, monthly family income per capita, payment for medical expenses, frequency of strokes, duration of disease, presence of somatic dysfunction, etc.

### Ethics approval:

The study was approved by the Institutional Ethics Committee of Baoding No.1 Central Hospital (No.: 2023095; date: October 23, 2023), and written informed consent was obtained from all participants.

### The Cognitive Appraisal of Health Scale:

A self-assessment scale to measure stroke patients’ cognitive appraisal of stroke events.^9^ In this study, Cronbach’s α coefficients were 0.75 for threat, 0.82 for challenge, 0.83 for beneficial/irrelevant, and 0.85 for harm/loss. The higher the score, the more inclined the individual is to appraise the event of having a stroke by this appraisal scale.

### Connor-Davidson Resilience Scale (CD-RISC-10):

A simplified version of the 10 items psychological resilience scale (CD-RISC-10).^10^,^11^ The higher the score, the better the individual’s psychological resilience.^12^ The Cronbach’s α coefficient was 0.94.

### Perceived Social Support Scale (PSSS):

To measure the perceived social support and level of family, friends and other people in stroke patients.^13^,^14^ The higher the score, the more social support is subjectively perceived by the individual. The total Cronbach’s α coefficient was 0.94.

### Medical Coping Modes Questionnaire:

To mainly assess the coping styles adopted by stroke patients when facing the disease.^15^ The Chinese version includes 20 items in three dimensions.^16^ With higher scores suggesting greater use of the corresponding coping style. The Cronbach’s α coefficients of this study were 0.793 for confrontation, 0.52 for avoidance, and 0.844 for submission.

### Stroke Stigma Scale:

To measure the level of stroke patients’ stigma.^17^ The higher the score, the greater the degree of stigma. The total Cronbach’s α coefficient was 0.880.

### Statistical analysis:

All data analyzed using the SPSS 25.0 software. The confidence interval was 95%. General data were expressed as the number of cases and percentages, and the chi-square test was used for comparison between groups. P-value<0.05 indicated a statistically significant difference. Pearson’s correlation analysis was taken for the correlation of influencing factors.

### Analysis of influencing factors:

The Logistic regression model and CART decision tree model were established to analyze the influencing factors of stigma, with stigma as the dependent variable and statistically significant general data, cognitive evaluation, coping style, psychological resilience and social support in the univariate analysis as the independent variables. The prediction efficiency of the two models was compared.

## RESULTS

The average age of the included 324 patients was 60.22±12.95 years old, of which 189 were male and 135 were female. The stigma score of stroke patients was (44.92±11.68) with a median of 46. The median stigma score was used to categorize the patients into two groups: high stigma risk group (≤46, assigned a value of 0) (160 cases, 49.4%) and low stigma risk group (>46, assigned a value of one) (164 cases, 50.6%). The results showed that the monthly family income per capita, dysfunction and self-care ability were statistically significant (*P*<0.05), Table-I.

**Table-I:** Univariate analysis of general data (*n*=324)

Item	Group	Low stigma risk group (*n*, %) (*n*=164)	High stigma risk group (*n*, %) (*n*=160)	^2^	P
Gender	Male	95 (57.93)	94 (58.75)	0.023	0.881
	Female	69 (42.07)	66 (41.25)		
Age (years)	<40	16 (9.76)	9 (5.63)	2.513	0.473
	40~	59 (35.98)	54 (33.75)		
	60~	80 (48.78)	88 (55.00)		
	≥80	9 (5.49)	9 (5.63)		
Marital status	Married	6 (3.66)	2 (1.25)	2.164	0.339
	Unmarried	149 (90.85)	147 (91.88)		
	Widowed	9 (5.49)	11 (6.88)		
Educational level	Elementary school or below	52 (31.71)	54 (33.75)	0.674	0.954
	Middle school	54 (84.38)	51 (31.88)		
	High school (Technical secondary school)	38 (23.17)	38 (23.75)		
	Junior college education or above	31 (18.90)	6 (3.75)		
Work status	Unemployed	20 (12.19)	24 (15.00)	3.647	0.456
	Employed	27 (16.46)	22 (13.75)		
	Retired	43 (26.22)	34 (21.25)		
	Engaged in agriculture	74 (45.12)	80 (50.00)		
Family residence	Urban	64 (39.02)	68 (42.50)	0.405	0.524
	Rural	100 (60.98)	92 (57.50)		
Monthly family income per capita (Yuan)	<2000	76 (46.34)	77 (48.13)	10.350	0.016
	2000~	65 (39.63)	54 (33.75)		
	4000~	16 (9.76)	27 (16.88)		
	≥6000	8 (4.88)	1 (0.63)		
Payment for medical expenses	Self-funded	13 (7.93)	6 (3.75)	2.947	0.400
	Employee’s Medical Insurance	56 (34.15)	59 (36.88)		
	Urban Health Insurance (New Cooperative Medical Scheme)	92 (56.09)	95 (59.38)		
	Commercial insurance	2 (1.22)	1 (0.63)		
Somatic dysfunction	Yes	115 (70.12)	74 (46.25)	18.988	0.000
	No	49 (29.88)	86 (53.75)		
Other chronic diseases	Yes	116 (70.73)	119 (74.38)	0.540	0.463
	No	48 (29.27)	41 (25.63)		
Self-care ability	Severe	15 (9.15)	2 (1.25)	20.674	0.000
	Moderate	105 (64.02)	92 (57.50)		
	Mild	44 (26.83)	58 (36.25)		
	Not required	0 (0)	8 (5.00)		
Duration of disease	<1	128 (78.05)	122 (76.25)	0.737	0.864
	1~	9 (5.49)	12 (7.50)		
	3~	12 (7.32)	10 (6.25)		
	≥5	15 (9.15)	16 (10.00)		
Frequency of strokes	<1	113 (68.90)	98 (61.25)	3.303	0.192
	2~	51 (31.09)	62 (38.75)		

Pearson’s correlation analysis showed that there was a statistically significant relationship between stigma and the influencing factors, Table-II. Logistic regression analysis results showed that patients with threat cognitive appraisal was risk factors for irrelevant cognitive appraisal, social support and psychological resilience, Table-III. Hosmer-lemeshow goodness-of-fit test *X^2^*=1.917, *df*=3, *P*=0.590, demonstrating good model fit.

**Table-II:** Correlation analysis of influential factors (*n*=342)

Variables	x±s	Challenge	Threat	Harm	Irrelevant	Secondary appraisal	Psychological resilience	Social support	Confrontation	Avoidance	Submission	Stigma
Challenge	19.72±4.47	1	-.553**	-.627**	.579**	.110*	.690**	.563**	.540**	-.375**	-.661**	-.637**
Threat	15.92±3.73	-.553**	1	.671**	-.439**	.074	-.534**	-.405**	-.323**	.359**	.506**	.580**
Harm	27.22±6.84	-.627**	.671**	1	-.604**	.149**	-.637**	-.406**	-.333**	.447**	.542**	.707**
Irrelevant	9.49±3.94	.579**	-.439**	-.604**	1	.065	.561**	.406**	.313**	-.348**	-.447**	-.569**
Secondary appraisal	17.05±2.09	.110*	.074	.149**	.065	1	-.008	.081	.078	.073	-.049	.065
Psychological resilience	21.91±8.81	.690**	-.534**	-.637**	.561**	-.008	1	.595**	.499**	-.476**	-.617**	-.667**
Social support	58.09±12.98	.563**	-.405**	-.406**	.406**	.081	.595**	1	.504**	-.275**	-.406**	-.507**
Confrontation	17.23±4.79	.540**	-.323**	-.333**	.313**	.078	.499**	.504**	1	-.364**	-.527**	-.389**
Avoidance	16.61±3.64	-.375**	.359**	.447**	-.348**	.073	-.476**	-.275**	-.364**	1	.445**	.462**
Submission	9.28±4.12	-.661**	.506**	.542**	-.447**	-.049	-.617**	-.406**	-.527**	.445**	1	.546**
Stigma	45.36±11.76	-.637**	.580**	.707**	-.569**	.065	-.667**	-.507**	-.389**	.462**	.546**	1

*Note:*

** indicates correlation significant at the 0.01 level (two-tailed);

* Indicates correlation significant at the 0.05 level (two-tailed).

**Table-III:** Results of binary Logistic regression analysis (*n*=342)

	B	S.E.	Wald	df	Significance	Exp (B)	95% confidence interval of Exp (B)	
							Lower limit	Upper limit
Dysfunction	-.305	.335	.828	1	.363	.737	.382	1.422
Self-care ability 1	-19.924	12237.898	.000	1	.999	.000	.000	.
Self-care ability 2	-19.849	12237.898	.000	1	.999	.000	.000	.
Self-care ability 3	-20.604	12237.898	.000	1	.999	.000	.000	.
Threat	1.021	.392	6.790	1	.009	2.776	1.288	5.982
Challenge	-1.088	.685	2.520	1	.112	.337	.088	1.291
Harm	.810	.474	2.924	1	.087	2.247	.888	5.684
Irrelevant	-1.137	.314	13.089	1	.000	.321	.173	.594
Social support	-2.319	.726	10.199	1	.001	.098	.024	.408
Avoidance	1.075	.787	1.865	1	.172	2.929	.627	13.697
Submission	-19.345	10436.874	.000	1	.999	.000	.000	.
Psychological resilience	-.825	.317	6.776	1	.009	.438	.236	.816
Constant	40.832	16083.977	.000	1	.998	5406E+17		

Cox and Snell R^2^: 0.411; Nagelkerke R^2^: 0.548. ^2^=171.594, P<0.001

The decision tree model results showed that 160 were at risk of high stigma, with an incidence of 49.4%. The most important independent variable affecting the decision tree target variable (stigma) was psychological resilience. When the score was ≤22, the incidence of high stigma risk was 73.3%. When the psychological resilience score was ≤22 and social support ≤61, the incidence of high stigma risk was 98%; When the psychological resilience score was ≤22, along with social support >61 and irrelevant cognitive appraisal ≤9, the incidence of high stigma risk was 73.8%. When the psychological resilience score was >22, along with threat cognitive appraisal >16 and irrelevant cognitive appraisal ≤9, the risk of high stigma was 50.0%.

**Fig.1:**
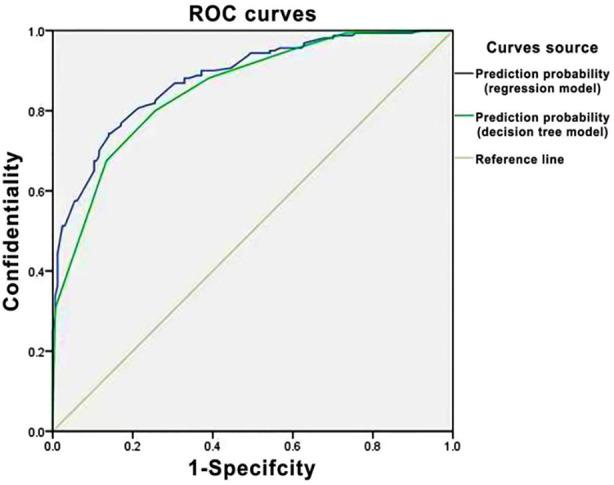
ROC curve.

The ROC curves of the two models were plotted. Fig.1 and the AUC was calculated, as shown in Table-IV. The results showed that the AUC of the decision tree model and the Logistic regression model were 0.854 and 0.880, and the accuracy respectively was 78.7% and 79.6%.

**Table-IV:** Comparison of classification effect between Logistic regression model and decision tree model (*n*=342)

Model	AUC	SD	P	95%CI	Accuracy (%)
Logistic regression model	0.880	0.018	0.000	0.844-0.916	79.6
Decision tree model	0.854	0.020	0.000	0.814-0.894	78.7

## DISCUSSION

Logistic regression analysis reveals a positive correlation between stigma and threat cognitive appraisal and a negative correlation with irrelevant cognitive appraisal in stroke patients. It indicates that threat cognitive appraisal is a risk factor for stigma in stroke patients and irrelevant cognitive appraisal is a protective factor. This is similar to the findings of national and international studies.^18^ Cognitive appraisal including primary appraisal, secondary appraisal and reappraisal. The primary appraisal is an appraisal of the event or situation itself, Secondary appraisal is mainly an appraisal of coping styles, coping resources, and so on. Reappraisal is an appraisal of the effectiveness and adaptability of one’s emotional and behavioral responses.^3^

The decision tree model shows that threat cognitive appraisal mediates the relationship between psychological resilience and stigma, through which patients’ stigma is reduced instead, suggesting a protective effect. This is in contrast to the results of the logistic regression analysis. The irrelevant cognitive appraisal is a diminished effect on stigmas compared to threat cognitive appraisal. It indicates that irrelevant cognitive appraisal mediates the relationship between social support and stigma, and that higher scores on irrelevant cognitive appraisal are associated with a lower incidence of stigma. Irrelevant cognitive appraisal is a protective factor for stigma.

The Logistic regression model and decision tree model illustrated that threat and irrelevant cognitive appraisals are predictors of stigma in stroke patients. It has been found that stigma is positively correlated with negative emotions, which exacerbates patients’ stigma by preventing them from fully understanding their illness and fearing the prognosis.^19^ Accordingly, patients’ health cognition should be understood in nursing care, and their positive cognition should be mobilized while their negative cognition should be minimized; at the same time, patients should be educated to accept the reality of suffering from a stroke event frankly, to enhance their confidence in overcoming the disease, and to alleviate their sense of stigma.

The Logistic regression analysis reveals a negative correlation between psychological resilience and stigma. The decision tree model shows that the influence of psychological resilience on stigma is located at the first tier of the decision tree, which indicates that psychological resilience is an important protective factor for stigma. A study found that psychological resilience in stroke patients was negatively correlated with negative emotions such as anxiety and depression.^20^ These studies have affirmed the important role played by patients’ psychological resilience during disease regression. Therefore, caregivers should analyze the psychological resilience level and characteristics of stroke patients, improve their psychological resilience level and promote their adaptation to stroke events, so as to promote their recovery. Logistic regression analysis reveals a negative correlation between social support and stigma, this is consistent with the findings of Ge C.^2^

The Logistic regression model and decision tree model have shown that social support is a major protective factor for stigma in stroke patients. Therefore, patients should be encouraged to actively seek social support networks in nursing care, while nursing workers should actively mobilize patients’ family members, friends and other people to care for, support and encourage patients, so as to improve their confidence in overcoming the disease and reduce their negative emotions of stigma. The decision tree model shows that dysfunction also has an effect on stigma, but it is not included in the Logistic regression analysis, suggesting that dysfunction has a weaker effect on stigma.

In the comparison of the two models, it is found that the Logistic regression model emphasizes more on the dependence between different influencing factors and stigma, it has higher accuracy and stability. But the decision tree model capable of visualizing and categorizing the influencing factors, providing a more reliable basis for targeted interventions.

### Limitations:

However, regression models and decision tree models are not included in the coping styles in this study, which may be related to the sample size or a single source of the sample. For this reason, the sample size needs to be enlarged and multicenter sampling should be adopted in future studies to make the results more reliable.

## CONCLUSIONS

The two models have their own advantages, combination of the advantages of the two models may be used while considering one of the higher accuracies of the two, in order to provide a new idea for the screening of predictors of stigma in stroke patients.

### Authors’ Contributions:

**WM and** **KJ** carried out the studies, data collection, drafted the manuscript, and are responsible and accountable for the accuracy or integrity of the work.

**RZ and XL:** Literature search, performed the statistical analysis and participated in its design.

**ZL** performed the statistical analysis, participated in its design. Critical Review.

All authors have read and approved the final manuscript.
